# Immune mediators in the tumor microenvironment of prostate cancer

**DOI:** 10.1186/s40880-017-0198-3

**Published:** 2017-03-14

**Authors:** Jinlu Dai, Yi Lu, Hernan Roca, Jill M. Keller, Jian Zhang, Laurie K. McCauley, Evan T. Keller

**Affiliations:** 1 Department of Urology and Biointerfaces Institute, University of Michigan, Ann Arbor, MI 48109 USA; 2 Center for Translational Medicine, Guangxi Medical University, Nanning, Guangxi 520021 P. R. China; 3 Periodontics and Oral Medicine, School of Dentistry, University of Michigan, Ann Arbor, MI 48109 USA; 4 Department of Urology, University of Michigan, Ann Arbor, MI 48109-8940 USA

**Keywords:** Prostate cancer, Tumor microenvironment, Macrophage, T-regulatory cell, Th17 cell, Interleukin-6, Receptor activator of nuclear factor kappa-B ligand

## Abstract

Prostate cancer tissue is composed of both cancer cells and host cells. The milieu of host components that compose the tumor is termed the tumor microenvironment (TME). Host cells can be those derived from the tissue in which the tumor originates (e.g., fibroblasts and endothelial cells) or those recruited, through chemotactic or other factors, to the tumor (e.g., circulating immune cells). Some immune cells are key players in the TME and represent a large proportion of non-tumor cells found within the tumor. Immune cells can have both anti-tumor and pro-tumor activity. In addition, crosstalk between prostate cancer cells and immune cells affects immune cell functions. In this review, we focus on immune cells and cytokines that contribute to tumor progression. We discuss T-regulatory and T helper 17 cells and macrophages as key modulators in prostate cancer progression. In addition, we discuss the roles of interleukin-6 and receptor activator of nuclear factor kappa-B ligand in modulating prostate cancer progression. This review highlights the concept that immune cells and cytokines offer a potentially promising target for prostate cancer therapy.

## Background

Prostate cancer tissues are composed of both tumor cells and host components. Host components consist of soluble factors (e.g., cytokines), stromal matrix, and cells. The milieu of host components in the tumor is termed the tumor microenvironment (TME) [1]. The cellular component of the TME consists of both host cells that are initially presented in a primary or metastatic lesion and cells that are recruited in response to either tumor- or host-derived factors. Most host cells, including stromal cells, vascular cells, and immune cells, may contribute to the function of the TME. Most cancer therapies directly target tumor cells through cytostatic or cytocidal activity. Recently, in addition to strategies targeting tumor cells, strategies targeting the TME have been explored. For example, it has been demonstrated that targeting the vasculature can reduce tumor-associated immunosuppression and thus improve therapeutic effects [2]. Accordingly, a thorough understanding of the TME is necessary to develop the most efficacious therapy.

Crosstalk between tumor cells and the TME results in an orchestrated evolution of both the TME and the tumor as the tumor progresses. The TME reacts to prostate cancer cell-produced soluble factors and directly interacts with prostate cancer cells. In return, the TME produces soluble factors, provides structural support, and contacts with cancer cells to influence the establishment and progression of prostate cancer [3]. There are several excellent reviews on tumor-TME crosstalk [4, 5]. In the current review, we focus on the immune component of the TME that promotes tumor progression.

### Inflammatory immune cells

Inflammation can potentially contribute to prostate cancer pathophysiology through several mechanisms, including generation of reactive oxygen species that lead to mutagenesis; production of cytokines to promote tumor growth and suppress anti-tumor immune response; and enhancement of the migration of tumor-promoting immune cells into the tumor [6, 7]. Immune cells compose the cellular arm of the inflammatory response, and in this review, we highlight some of these cells and how they have been thought to contribute to prostate cancer progression (Table 1).

**Table 1:** Summary of key immune cells and cytokines found in the tumor microenvironment of prostate cancer

Type	Prevalence and function	References
Treg	Definition is constantly refined; however, FoxP3 is consistently expressed. Highly prevalent in prostate cancer tissues. Potential roles include immunosuppression, allowing tumors to escape from immunosurveillance	[9, 11, 13, 84]
Th17 cells	CD4^+^ T cells that express high levels of IL-17. The number of Th17 cells in tumors inversely associates with tumor grade, and the number of Th17 cells in peripheral blood inversely correlates with time to progression in patients with prostate cancer	[10, 17, 18]
Macrophages	Recruited to tumors by chemotactic factors such as CCL2. Produce a variety of pro-inflammatory factors that promote angiogenesis and tumor growth. The increased amount of tumor-infiltrating macrophages directly associates with an unfavorable prognosis	[85–88]
IL-6	A pro-inflammatory cytokine. Elevated levels of IL-6 are associated with prostate cancer biochemical recurrence. Promote prostate cancer cell growth in vitro and in vivo	[40, 42, 54]
RANKL	Produced by activated T cells and osteocytes. Target dendritic cells to promote antigen presentation. Tumors can directly produce RANKL or induce RANKL expression from the TME, resulting in osteoclastogenesis and tumor-induced bone resorption	[58, 60, 72, 83]

*CCL2* chemokine (C–C motif) ligand-2, *IL-6* interleukin-6, *IL-17* interleukin-17, *RANKL* receptor activator of nuclear factor kappa-B ligand, *Th17* T helper 17, *Treg* T-regulatory cell, *TME* tumor microenvironment, *FoxP3* forkhead box P3

#### Tumor-modulating T cells and prostate cancer

Tumor-infiltrating lymphocytes (TILs) contribute to the progression of prostate cancer through multiple mechanisms that have not been well defined yet. One type of TIL, the T-regulatory cell (Treg), has been identified as a primary mediator in cancer progression. Tregs are CD4^+^ T cells that inhibit the activity of T effector cells; however, specific definitions based on cell surface markers constantly change due to heterogeneity of the Treg population [8]. This constant evolution of defining the Treg phenotype creates a challenge in interpreting the literature, and one must be aware of how the Treg is defined in a particular publication. Tregs tend to suppress anti-tumor responses rather than promote tumor cell growth directly. In support of the importance of Tregs in prostate cancer, Tregs that were defined as CD4^+^CD25^high^ cells with in vitro immunosuppressive function were found to be increased in prostate cancer tissues compared with non-cancerous prostate tissues [9]. In addition, to determine the phenotype of TILs, Sfanos et al. [10] performed multiple biopsies of prostate cancers and determined the phenotypes of the procured cells by flow cytometry. They found that tumor-infiltrating CD4^+^ T cells were skewed toward Treg (FoxP3^+^, forkhead box P3) and T helper 17 (Th17) phenotypes rather than Th2 phenotype. This finding was extended to the peripheral blood, where the proportions of CD4^+^CD25^high^ Tregs were increased in men with prostate cancer compared with healthy donors [9]. Further studies showed that tumor-infiltrating Tregs, defined as CD8^+^ FoxP3^+^ cells, suppressed naive T cell proliferation mainly through a cell contact-dependent mechanism [11]. However, the ability to suppress T-cell proliferation may not always result in inhibition of tumor-specific immune activity. In a transgenic adenocarcinoma mouse prostate (TRAMP) model of prostate cancer, CD4^+^CD25^+^FoxP3^+^ Tregs were found to be dispensable for induction of tumor-specific tolerance [12]. Yokokawa et al. [13] evaluated levels of CD4^+^CD25^high^FoxP3^+^ Tregs in the peripheral blood of healthy donors and patients with biochemically progressive, localized, or metastatic prostate cancer by flow cytometry. The function of Tregs was determined by their ability to suppress the proliferation of CD4^+^CD25^−^ T cells [13]. They found that although no differences were observed in the amount of Tregs in the peripheral blood among different groups, Tregs from patients with prostate cancer had significantly greater suppressive function than Tregs from healthy donors [13]. In addition to the tumor itself, cancer therapies may also affect Treg functions. For example, androgen modulation is an important and often-used therapy for prostate cancer. In a mouse model, androgen ablation induced a transient increase of CD4^+^ T cells and CD8^+^ T cells in residual tumor [14]. More than 2 months later, FoxP3^+^ Tregs were increasingly found within prostate epithelium, whereas cytotoxic T lymphocytes, which were evenly distributed before androgen ablation, became sequestered within stroma [14]. Thus, androgen modulation could affect the efficacy of an immunotherapy regimen and should be considered when patients with prostate cancer are treated with immune-dependent regimens. Taken together, these results indicate tumors and tumor therapies can affect Treg function; however, large clinical trials to validate these findings should be performed.

In addition to Tregs, another subset of CD4^+^ T cells, Th17 cells, may affect prostate cancer biology and responses to immunotherapy. Th17 cells are CD4^+^ T effector cells that produce a large amount of interleukin-17 (IL-17) which is a pro-inflammatory cytokine that attracts and activates granulocytes and monocytes [15]. The role of Th17 cells in cancer is controversial. They have been reported to both inhibit cancer and promote it [16]. An initial clue to the importance of Th17 cells came from an early study which demonstrated that injection of a fusogenic glycoprotein in combination with heat shock protein 70 (HSP70), as an immune adjuvant, induced an anti-tumor T-cell immune response that was associated with increased IL-17 expression [17]. To compare the prognostic implications of the pretreatment level of Th17 cells with those of Tregs in prostate cancer patients who received active whole-cell vaccine-based immunotherapy, Derhovanessian et al. [18] detected the frequencies of Th17 cells and Tregs in the peripheral blood of patients with hormone-resistant non-bone metastatic prostate cancer prior to immunotherapy. They found that the frequency of Th17 cells inversely correlated with time to disease progression. Furthermore, patients who responded to immunotherapy with significant reductions in prostate-specific antigen (PSA) velocity showed a Th17 profile similar to that of healthy men; whereas, those who did not respond had a significantly different Th17 profile compared with that of responders and healthy men [18]. In contrast, although the frequency of Tregs in the peripheral blood was higher in men with prostate cancer than in age-matched healthy men, no difference was observed between responders and non-responders [18]. These data indicated that Th17 cells may predict therapeutic response to active whole-cell vaccine-based immunotherapy. The true role that Th17 cells play in prostate cancer (i.e., inhibit or promote prostate cancer progression) may depend on tumor stage. Additional research efforts should be devoted to this concept.

#### Macrophages and prostate cancer

Macrophages have been found to play intriguing roles in promoting tumor progression. Among bone marrow-derived cells in the TME, macrophages are one type of these cells that significantly associate with tumor progression and immunosuppression [19–23]. It has been well recognized that macrophages infiltrate the tumor tissue (these macrophages are termed tumor-associated macrophages [TAMs]); moreover, for prostate cancer, more macrophages were observed in metastatic nodules than in primary tumors [24]. The increase of TAMs in prostate cancers may be mediated through prostate cancer-derived parathyroid hormone-related protein (PTHrP) which has been shown to recruit myeloid cells via osteoblast-produced chemokine (C–C motif) ligand 2 (CCL2) [25]. Although an increase of TAMs in prostate cancers is well recognized, little is known about the specific mechanisms by which macrophages promote tumor growth.

A primary role of macrophages is phagocytosis during bacterial infection [26], yet phagocytosis is an often overlooked function relative to tumorigenesis [27, 28]. Efferocytosis refers to the specific phagocytosis of apoptotic cells and is an integral process in tissue homeostasis, inflammation, and autoimmunity [29]. Like many other tumor-promoting activities, efferocytosis is a physiologic activity that may be hijacked by tumors to benefit their establishment and growth.

Macrophages perform efferocytosis via distinct receptor signaling pathways [30]. Specific “eat me” signal molecules, such as milk fat globule-E8 (MFG-E8), are expressed by activated macrophages and bridge apoptotic cells and macrophages to facilitate efferocytosis [31–33]. MFG-E8 includes the N-terminus that bears a signal peptide to direct secretion, an epidermal growth factor (EGF)-repeated domain that contains an Arg-Gly-Asp (RGD) motif for recognition of integrin on phagocytic cells, and the C-terminus that bears a factor V/VIII-like domain enabling binding to phosphatidylserine in apoptotic cells [33]. MFG-E8 is produced by several types of cells, most notably macrophages, and co-localizes with the activated marker CD68 [34]. The majority of research on MFG-E8 has been performed in the mammary gland, where it was originally identified, as well as in the peritoneal cavity, spleen, and lung [34]. In patients with triple-negative breast cancer, MFG-E8 promotes breast cancer progression through the p63 pathway; but in estrogen receptor- and erbB2-positive breast cancers, MFG-E8 serves a suppressive function [35]. In the context of prostate cancer bone metastasis, TAMs are polarized upon interaction with apoptotic tumor cells in an MFG-E8-dependent manner that supports tumor progression [36], similar to that observed in triple-negative breast cancer [35]. In the bone microenvironment, factors released by efferocytic macrophages could increase the resistance of bone metastases to cancer therapies, resulting in a lack of therapeutic response or reduced response duration. MFG-E8 and other intra- and extra-cellular signaling events specifically related to macrophage-mediated efferocytosis represent intriguing new pharmacologic targets for patients with bone metastasis.

### Cytokines and prostate cancer

A variety of cytokines are secreted by cells in the TME that can affect prostate cancer growth. These cytokines can act in a paracrine fashion on tumor cells to stimulate a variety of tumor activities, including proliferation, chemoresistance, anti-apoptosis, migration, and invasion. In this review, we focus on two immune-related cytokines, interleukin-6 (IL-6) and receptor activator of nuclear factor kappa-B ligand (RANKL), that are expressed in the TME of prostate cancer.

#### IL-6

IL-6 is produced by inflammatory cells and osteoblasts and has ample opportunities to interact with prostate cancer cells [37]. A great deal of clinical and experimental evidence suggests that IL-6 promotes prostate cancer progression [38]. Several studies have shown that IL-6 level is elevated in the sera of patients with metastatic prostate cancer [39–41]. For example, Alcover et al. [40] assessed serum levels of IL-6 and its soluble receptor to determine if these levels could be used to predict biochemical recurrence in patients who underwent radical prostatectomy. They found that preoperative serum levels of IL-6 higher than 1.2 pg/mL in men with prostate cancer were associated with an increased probability of biochemical recurrence (i.e., increased serum PSA levels) [40]. Although the study had a small sample size, the results support further evaluation of IL-6 as a prognostic factor. Similarly, Stark et al. [41] proved that pre-diagnostic IL-6 level was associated with time to progression/death for prostate cancer patients with healthy weight. Overall, the preponderance of evidence suggests that IL-6 level is elevated in men with prostate cancer and is related to the clinical outcome of prostate cancer.

In addition to these clinical observations, in vitro cellular studies have shown that IL-6 modulates the growth of prostate cancer cells. Chung et al. [42] showed that IL-6 promoted the growth of hormone-refractory cells but had no effect on hormone-dependent cell lines. Some studies have reported that the addition of exogenous IL-6 to the culture media of prostate cancer LNCaP cells resulted in a dose-dependent growth inhibition with neuroendocrine differentiation [43, 44], whereas in other instances, cell proliferation was increased [42–48]. The reasons for these differences have not yet been clarified, and it appeared that IL-6 inhibited the growth of only LNCaP cells but not other prostate cancer cell lines [49].

In addition to increased proliferation, decreased apoptosis can also promote tumor growth. IL-6 has an anti-apoptotic effect on many types of cancer cells, including prostate cancer cells [50, 51]. A previous study demonstrated that orchiectomy induced a conversion of LuCaP 35 tumors to an androgen-independent phenotype through increased IL-6 expression [52]. It has been shown that targeting IL-6 with an IL-6 antibody promoted the apoptosis of androgen-independent PC-3 cells in mice [53]. IL-6 protects prostate cancer cells against apoptosis through activation of signal transducer and activator of transcription 3 (STAT3) [50] and phosphatidylinositol-3 kinase (PI3K) [51]. Taken together, decreased proliferation and increased apoptosis caused by inhibiting IL-6 suggest that IL-6 coordinates both processes to promote the growth of androgen-independent tumors.

In addition to the effect of enhancing cell proliferation, IL-6 also enhances other aspects of prostate cancer. For example, IL-6-mediated activation of STAT3 activates insulin-like type I growth factor receptor (IGF-IR), resulting in tumorigenesis [54]. IL-6 is also associated with neuroendocrine differentiation of prostate cancer cells [44]. In combination with the cytokine CCL2, IL-6 induces M2-type macrophage polarization and promotes CD11b^+^ peripheral blood mononuclear cell survival [55]. Together, these findings indicate that IL-6 contributes to prostate cancer progression through multiple activities.

The above published studies suggesting the contribution of IL-6 to prostate cancer progression provided the rationale for a clinical trial to evaluate IL-6-targeted therapies. A human-mouse chimeric monoclonal neutralizing IL-6 antibody (siltuximab, also known as CNTO 328) has been evaluated in a phase II study in men with advanced castration-resistant prostate cancer, with PSA response rate being the primary endpoint [56]. However, the response rate was only 3.8%, and no men with measurable disease had a response according to the Response Evaluation Criteria in Solid Tumors (RECIST); even so, C-reactive protein, an indicator of IL-6 activity, was decreased, suggesting that IL-6 was effectively neutralized [56]. These results suggest that targeting IL-6 in men with advanced castration-resistant disease may not result in a significant direct anti-tumor benefit. In contrast to a direct anti-tumor effect, IL-6 has an anti-apoptotic effect [57], suggesting a possibility that targeting IL-6, which would block the anti-apoptotic effect of IL-6, may synergize with a chemotherapy regimen by promoting cell death.

#### RANKL

RANKL is expressed on the surface of activated T cells and interacts with its receptor, receptor activator of nuclear factor kappa-B (RANK), on dendritic cells (DCs) to promote the survival of DCs and increase their activities as antigen-presenting cells [58]. In addition to its direct immune function, RANKL promotes osteoclastogenesis. It has been identified that osteocytes also produce RANKL in the bone microenvironment [59]. RANKL can be expressed as a membrane-bound molecule or, in some instances, be cleaved to form a functional soluble RANKL peptide [60]. RANKL binds to the transmembrane receptor RANK on osteoclast precursors and initiates osteoclastogenesis [61, 62]. It has been demonstrated that RANKL and RANK are required for osteoclastogenesis since transgenic *rankl* ^−/−^ and *rank* ^−/−^ mice had no osteoclasts or developed osteosclerotic bones [63, 64]. Osteoprotegrin (OPG) is a soluble glycoprotein that negatively regulates osteoclastogenesis through sequestering RANKL, resulting in the block of its interaction with RANK [65]. Thus, the balance between RANKL and OPG determines the extent of bone resorption.

Many cancers found in the skeleton [66], including osteoclastomas [67] and prostate cancer bone metastases [68], have dysregulated RANKL, OPG, or RANK expression. Both prostate cancer epithelium and stromal cells express RANKL [68], whose level directly associates with disease stage [69] and may have a prognosis-predictive value [70]. Prostate cancer cells initiate osteoclastogenesis through RANKL [71, 72]. In murine models of prostate cancer bone metastasis, using OPG [71] or soluble RANK [73] to inhibit RANKL activity decreased both the amount of mature osteoclasts and the frequency of bone lesions. The decrease of tumor-induced bone destruction is associated with decreased pain-associated behaviors in murine models of bone cancer [74, 75]. Taken together, these findings suggest that inhibiting RANKL will diminish both tumor-associated bone remodeling and bone pain.

In addition to its effect on bone remodeling, RANKL has direct effects on cancer cells. For example, RANKL binds with RANK on prostate cancer cells and then induces pro-metastatic gene expression, resulting in increased invasive ability of prostate cancer cells [76]. Furthermore, inhibition of RANKL by OPG promotes prostate cancer cell survival through inhibition of TNF-related apoptosis-inducing ligand (TRAIL)-mediated apoptosis [77]. These findings, in combination with the reports of tumor-mediated bone resorption via RANKL, indicate that targeting RANKL would have a two-pronged therapeutic effect by inhibiting both tumor-induced bone resorption and pro-metastatic activity of tumor, which have provided the rationale to evaluate the clinical efficacy of targeting RANKL in cancer-related bone diseases.

The anti-tumor effect of denosumab, a human monoclonal IgG2 antibody that targets RANKL, has been evaluated in several clinical trials. Denosumab reduced the expression of urinary N-telopeptides of collagen (uNTx), a biomarker for osteoclast activity, in a phase II study that included patients with prostate cancer bone metastases [78]. In another clinical trial, bone mineral density (BMD) was significantly improved compared with baseline after treatment with denosumab in men with prostate cancer who were being treated with androgen-deprivation therapy [79, 80]. Also in a clinical trial, zoledronic acid (an anti-osteoclastic bisphosphonate) was compared with denosumab for prevention of skeletal-related events (SREs: defined as presence of bone fracture, need for bone pain palliation, or need for bone surgery) in men with castration-resistant prostate cancer and bone metastases [81]. Denosumab significantly delayed the time to first on-study SRE as well as the time to first and subsequent on-study SRE compared with zoledronic acid [81]. The above studies and additional preclinical and clinical data [82, 83] have supported the United States Food and Drug Administration approval of denosumab for treating patients with prostate cancer (and breast cancer) bone metastases.

## Conclusions and future directions

**Fig. 1:**
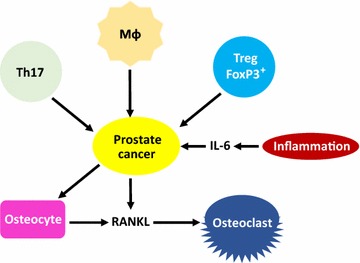
Key immune cells and cytokines in the tumor microenvironment of prostate cancer. Tregs suppress the activation of anti-tumor T cells to promote tumor progression. Th17 cells may promote or inhibit prostate cancer progression depending on the context. Macrophages promote prostate cancer growth through efferocytosis. Inflammatory stimuli can lead to IL-6 production, which, in turn, can promote prostate cancer growth. Prostate cancer cells can produce RANKL directly and stimulate host cells (e.g., osteocytes) to produce RANKL in the tumor microenvironment. RANKL can then mediate tumor-induced bone remodeling through osteoclast activation. *IL-6* interleukin-6, *Mɸ* macrophage, *RANKL* receptor activator of nuclear factor kappa-B ligand, *Th17* T helper 17, *Treg* T-regulatory cell, *FoxP3* forkhead box P3

Multiple host factors in the TME contribute to prostate cancer progression (Fig. 1; Table 1). Immune cells, such as Tregs, Th17 cells, and macrophages, are major modulators of prostate cancer progression. In addition, host cells affect prostate cancer cells via cytokines. Cytokines such as IL-6 and RANKL have pleiotropic actions on prostate cancer cells. These findings show that the TME offers potentially promising targets for prostate cancer therapy. Further studies on host immune factors, in addition to studies on tumors, may validate their potential therapeutic benefits.
